# UV–Vis Detection of Thioacetamide: Balancing the Performances of a Mn(III)-Porphyrin, Gold Colloid, and Their Complex for Selecting the Most Sensitive Material

**DOI:** 10.3390/mi16050574

**Published:** 2025-05-14

**Authors:** Camelia Epuran, Ion Fratilescu, Ionela Fringu, Anca Lascu, Liliana Halip, Mihaela Gherban, Eugenia Fagadar-Cosma

**Affiliations:** 1 Institute of Chemistry “Coriolan Dragulescu”, Mihai Viteazu Avenue 24, 300223 Timisoara, Romania; ecamelia@acad-icht.tm.edu.ro (C.E.); alascu@acad-icht.tm.edu.ro (A.L.); lili.ostopovici@acad-icht.tm.edu.ro (L.H.); 2 National Institute for Research and Development in Electrochemistry and Condensed Matter, P. Andronescu Street, No. 1, 300224 Timisoara, Romania; mihaelabirdeanu@gmail.com; 3 Romanian Academy, School of Advanced Doctoral Studies of the Romanian Academy (SCOSAAR), Institute of Chemistry “Coriolan Dragulescu”, Mihai Viteazu Avenue 24, 300223 Timisoara, Romania

**Keywords:** manganese porphyrin, UV–Vis sensors, gold nanoparticles, porphyrin–AuNP complex, thioacetamide detection

## Abstract

The optical detection of thioacetamide was investigated using a metalated porphyrin, Mn(III)-5,10,15,20-tetrakis-(3,4-dimethoxyphenyl)-21H,23H-porphyrin chloride (Mn-3,4-diMeOPP), a gold colloid solution (AuNPs), and a complex formed between them (Mn-3,4-diMeOPP–AuNPs) in order to select the most sensitive material and to achieve complementarity between methods. Mn-3,4-diMeOPP, AuNPs, and their complex were synthesized and characterized by means of UV–Vis, FT-IR spectrometry, and AFM investigations. It could be concluded that Mn-3,4-diMeOPP could detect/quantify thioacetamide (TAA) in the range 3.13 × 10^−8^ M–7.67 × 10^−7^ M in a linear fashion, with a 99.85% confidence coefficient. The gold colloidal particles alone could detect TAA in an extremely narrow concentration domain of 2–9.8 × 10^−7^ M, slightly complementary with that of Mn-3,4-diMeOPP. The complex between Mn-3,4-diMeOPP and gold colloid proved to be able to quantify TAA in the trace domain with concentrations of 1.99 × 10^−8^ M–1.76 × 10^−7^ M in a polynomial fashion, with the method being more difficult. A potential mechanism for TAA detection based on Mn-3,4-diMeOPP is discussed based on computational modeling. The distorted porphyrin conformation and its electronic configuration favor the generation of a grid of electrostatic interactions between porphyrin and TAA.

## 1. Introduction

The versatile reactivity of manganese porphyrins is based on the one hand, on the porphyrin moiety and, on the other hand, on the metal center, due to both their ability to coordinate different axial ligands and their redox capabilities [1]. Furthermore, based on the fact that Mn porphyrins evidence nonplanarity of the porphyrin ring systems [2], these metallo-structures are frequently employed in the formulation of optical [3], chemical [4], and electrochemical sensors [5]. Manganese porphyrins have been shown to interact with sulfur-containing compounds, thus oxidizing hydrogen sulfide (H_2_S) and forming polysulfides [6] to catalyze protein S-glutathionylation [7] and to affect intracellular sulfide metabolism [8].

On the other hand, gold colloids and AuNPs are also widely used as sensing compounds for toxic [9], biologically active [10], and pharmaceutical compounds [11,12,13]. Nano-gold plasmonic systems are often connected with improved performance in analyte detection, often associated with the benefit of requiring lower volumes/concentrations of the samples for detection [14]. The sensitivity of analyte detection provided by AuNPs, high above that of standard optical biosensing, is due to both their chemical stability and their ability to support excitation of localized surface plasmon resonances (LSPRs) in a wide wavelength range from visible [15] to mid-infrared [16]. Recently, an excellent approach led to the creation of an optofluidic sensor based on antibody-functionalized gold, which was capable of recognizing and precisely quantifying different species of α-synuclein protein, connected with some neurodegenerative diseases, such as Parkinson’s and Alzheimer’s [17]. Based on our previous experience, the association between gold nanoparticles (AuNPs) and suitable partners, such as 5,10,15,20-tetrakis-(4-methylphenyl)porphyrinato manganese(III) chloride and Co(II)-5,10,15,20-meso-tetra(3-hydroxyphenyl)porphyrin, used due to their plasmonic properties and high surface-to-volume ratio, is viable for the detection of various analytes such as β-carotene [18], ascorbic acid [19], chloramphenicol [20], and procaine [21].

Thioacetamide (TAA) is a sulfur-containing compound widely used in the laboratory and in various technical applications, such as fungicides [22], rubber chemicals [23], curing agents [24], cross-linking agents [25], metallurgy [26], and pesticides [27]. Unfortunately, in spite of its benefits, TAA causes liver, kidney, and bone damage [28]. It was demonstrated that TAA given at a dose of 50 mg/kg/day, representing 6.65 × 10^−4^ mol/kg, intraperitoneally administrated for 5 days/week over two weeks [29], caused kidney injuries. In addition, TAA is a known liver toxicant that promotes fibrosis. In an in vitro study in rats, by administering a small 0.025 mM concentration, primary hepatocytes were produced [30,31]. The range recommended for studying liver injury due to the induction of liver toxicity [32] in various strains of mice, with effects on potential regeneration, is from 300 to 800 mg/kg. Doses beyond 900 mg/kg are lethal in most mouse strains [33]. The low-dose group showed that rats treated with 200 mg/Kg TAA suffered modifications to their bone surface, tissue surface, and bone volume, especially the cortical bone and trabecular bone. In addition, the bones of rats tested with an intraperitoneal injection of TAA could resist less pressure and were prone to fractures [34].

These toxicity levels are the reason why the monitoring of TAA concentrations is considered essential. An overview of the recent literature shows a few thioacetamide detection methods based on electrochemical methods, especially voltammetry [35,36,37], triboelectric sensing [38], and electrochemiluminescence and luminescence [39,40], which are presented in Section 3 (Table 1) along with their performances and intrinsic shortcomings/benefits compared to the results of our work.

**Figure 1:**
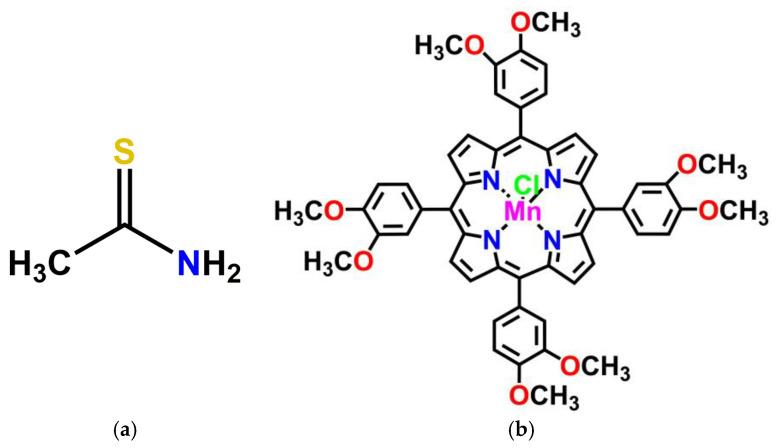
Structures of (**a**) thioacetamide and (**b**) Mn(III)-5,10,15,20-tetrakis-(3,4-dimethoxyphenyl)porphyrin chloride (Mn-3,4-diMeOPP).

Our purpose was to develop a fast and facile UV–Vis optical method to detect thioacetamide (Figure 1a) and to quantify its concentration by using as a sensing substance either Mn(III)-5,10,15,20-tetrakis-(3,4-dimethoxyphenyl)porphyrin chloride (Mn-3,4-diMeOPP) (Figure 1b), AuNPs, or a complex formed between Mn-3,4-diMeOPP and AuNPs and leveraging the obtained results to decide which material was best-suited to the UV–Vis detection of TAA.

## 2. Materials and Methods

### 2.1. Reagents

The 3,4-dimethoxybenzaldehyde, and pyrrole originated from Sigma-Aldrich, St Louis, MO, USA, glucose (Glu), NaCl, urea, ascorbic acid (AA), and ammonium oxalate (AmOxa) were obtained from Chimreactiv/Reactivul (Bucharest, Romania), dichloromethane, 2,3-dichloro-5,6-dicyano-1,4-benzoquinone, BF_3_·OEt_2_, manganese(II)chloride, sodium sulfate, sodium acetate (SA), sodium salicylate (SS), calcium gluconate (CaG), KI, and KCl were procured from Merck, Darmstadt, Germany, and tetrahydrofurane (THF) and FeCl_3_ were obtained from Fluka Chemie Bucks, Switzerland. All reagents were of purum analiticum quality and used as received.

#### 2.1.1. Synthesis of 5,10,15,20-Tetrakis(3,4-dimethoxyphenyl)-21H,23H-porphyrin (3,4-diMeOPP)

The porphyrin base was previously synthesized and completely characterized [41], as follows: a mixed equimolar solution containing 0.298 g (1.8 mmol) 3,4-dimethoxybenzaldehyde and 0.13 mL (1.8 mmol) pyrrole, in 300 mL of distilled CH_2_Cl_2_, was purged with N_2_ for 15 min. Then, 0.3 mL of BF_3_ OEt_2_ was added at room temperature. The solution was left during 3 h and, afterward, neutralized with triethylamine. Subsequently, 0.8 g (3.5 mmol) 2,3-dichloro-5,6-dicyano-1,4-benzoquinone (DDQ) in 40 mL of toluene was added and the reaction mixture was left overnight. The solvent was removed, and the residue was chromatographed on alumina (eluents CH_2_Cl_2_/petroleum ether 40/60 (*v*/*v*)), giving the porphyrin as a red band. Recrystallization using CH_2_Cl_2_/methanol gave the desired dark-red compound (η = 19.4%) [42].

#### 2.1.2. Obtaining of Mn(III)-5,10,15,20-Tetrakis-(3,4-dimethoxyphenyl)porphyrin Chloride (Mn-3,4-diMeOPP)

The synthesis of Mn-3,4-diMeOPP was performed using the classical metalation procedure, by reacting 0.6 g (0.7 mmol) 5,10,15,20-tetrakis-(3,4-dimethoxyphenyl)-21H,23H-porphyrin, dissolved in 200 mL dimethylformamide, with 0.453 g (2.8 mmol) MnCl_2_ dissolved in 10 mL methanol, at refluxing temperature, for 1 h. The resulting reaction mixture was dried by vacuum evaporation; the resulting solid was then dissolved in dichloromethane and repeatedly washed with distilled water. The organic extracts were dried with anhydrous sodium sulfate and concentrated. The yield was approximately 80%, obtained after recrystallization and final purification by column chromatography on silica gel using tetrahydrofuran as an eluent. As the manganese (III) porphyrins were paramagnetic and showed nuclear magnetic signals too broad to be detected, we did not perform ^1^H-NMR analysis on the newly obtained Mn(III)-metalated porphyrin [43,44,45].

#### 2.1.3. Method for Gold Colloid Preparation

The gold colloidal solution was prepared using a previously published obtaining method [46], which uses HAuCl_4_·3H_2_O and sodium citrate. The concentration of AuNPs obtained was 8.37 × 10^−4^ M.

#### 2.1.4. Method for Obtaining the Mn-3,4-diMeOPP-AuNP Complex

To a 5 mL solution of Mn-3,4-diMeOPP in THF (c = 3.180 × 10^−5^ M), different portions of gold colloidal solution (c = 5 × 10^−4^ M) were added. After each addition, stirring was maintained for 90 s and then the UV–Vis spectrum was recorded, which is discussed in Results Section 3.3. Complexation took place until a mole ratio for Mn-3,4-diMeOPP/AuNPs of 1/4 was reached.

### 2.2. Apparatus

The UV–Vis spectra were recorded on a V-650 JASCO spectrometer (Pfungstadt, Germany), using 1 cm wide quartz cuvettes. To obtain atomic force microscopy (AFM) images, a Nanosurf^®^ EasyScan 2 Advanced Research AFM microscope (Liestal, Switzerland) equipped with a piezoelectric ceramic cantilever was used. A SXZ3-1198 Agilent FTIR Cary 630 Spectrometer obtained from Agilent Technologies (Santa Clara, CA, USA, 2015) produced the infrared spectra of the samples, prepared as KBr pellets.

### 2.3. Detection Method for Thioacetamide by UV–Vis Method Using Mn-3,4-diMeOPP as Sensitive Substance

To a 5 mL solution of Mn-3,4-diMeOPP (c = 3.180 × 10^−5^ M) in THF, successive portions were added consisting of 0.02 mL TAA solution in water (c = 1 × 10^−5^ M). After each addition of TAA, the mixture was stirred for 1 min, and then the UV–Vis spectrum was recorded for the THF/water mixture. The discussion of the obtained data is presented in Results Section 3.2.

### 2.4. Optical Detection of Thioacetamide Using Gold Colloid as Sensitive Material

The interaction between the thioacetamide (c = 1 × 10^−5^ M) and AuNPs was performed as follows: to 5 mL AuNP solution in water, portions of 0.04 mL thioacetamide solution in water (acidified with HCl to pH = 1.5) were continuously added. For each addition of TAA, the solution mixtures were stirred for 90 s and the UV–Vis spectra were recorded. The discussion is presented in Results Section 3.3.

## 3. Results and Discussion

### 3.1. Comparative Study of Physical–Chemical Characteristics of 3,4-diMeOPP and Mn-3,4-diMeOPP

#### 3.1.1. Comparison of UV–Vis Spectra

**Figure 2:**
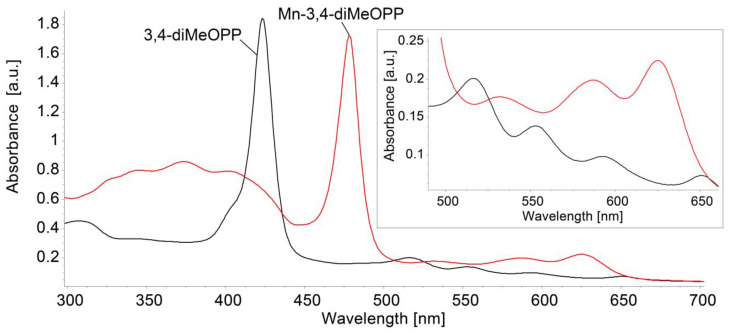
Superimposed UV–Vis spectra for 3,4-diMeOPP and Mn-3,4-diMeOPP in THF, performed at the same concentration of 3.392 × 10^−5^ M. In detail, the allure and absorption range of the Q bands are magnified.

The UV–Vis spectrum of 3,4-diMeOPP was previously presented and characterized [41]. Figure 2 shows the overlapping electronic spectra of the porphyrin base and the manganese-metalloporphyrin. The Soret band located at 421 nm in the case of the porphyrin base is visibly shifted to 478 nm in the case of Mn-3,4-diMeOPP. A decrease in the number of Q bands for Mn-metalloporphyrin can be observed, accompanied by both blue shifting and a high augmentation in the intensity of the QI band. This is due to the increase in symmetry of the metalated porphyrin to D_4h_, compared to only D_2h_ symmetry existing in the porphyrin base. The UV–Vis spectrum of the Mn(III) porphyrin presents significant metal–porphyrin π interactions, where the metal ion is coordinated in the center of the macrocycle. Due to having unoccupied orbitals in the metal, all manganese porphyrins exhibit e_g_(d(π))–d_yz_ and d_xz_ symmetries, a d-hyper-type absorption spectrum, characterized by the appearance of three extra B bands in the 320–450 nm region, which can be seen in Figure 2 [47,48,49]. The intensity of the Q bands (assigned to a_2u_(π)–e_g_^*^(π) transitions) is often correlated with the stability of any metalloporphyrin: if the Q bands are displayed with low intensity, then the metalloporphyrin is considered highly stable and its atoms are probably located in the square plane [50]. The coordination between the Mn(III) ion and nitrogen atoms in the porphyrin inner core might extend the conjugation from the porphyrin to the metal ion; in our case, electronic excitation requires lower energy absorption and, consequently, a longer wavelength than the pure porphyrin [51].

#### 3.1.2. FT-IR Analysis

**Figure 3:**
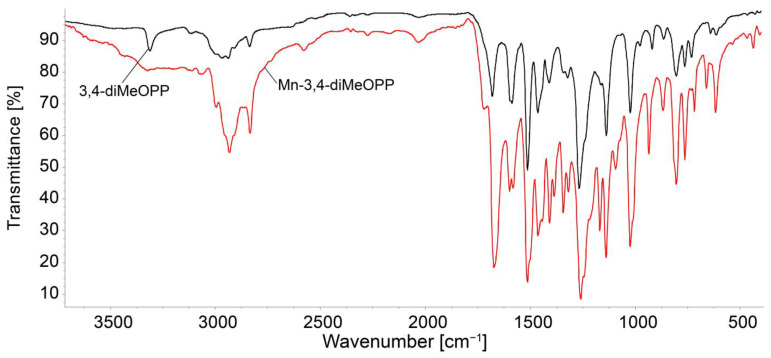
Comparative FT-IR spectra for 3,4-diMeOPP and Mn-3,4-diMeOPP from KBr pellets.

The overlapping FT-IR of the porphyrin base and Mn-metalloporphyrin are presented in Figure 3. Common features of the FT-IR spectra include the stretching vibration of the C–H_pyrrol_ bond at 802 cm^−1^ and its bending vibration, located both at 1023 and around 1139 cm^−1^. The C-N bond is present in both spectra at around 1260 cm^−1^. The band at 1460 cm^−1^ can be attributed to the C=N bond stretching vibration. The signals located at 763 cm^−1^ and 2835 cm^−1^ represent the bending and stretching vibrations of the C-H_phenyl_ bonds, respectively [41]. The N-H bond characteristic for the porphyrin-base, located around 3345 cm^−1^ [52], is no longer present in the spectrum of the metalated porphyrin, proving that the metal ion is linked to the nitrogen atoms in the porphyrin core. The Mn-N bond vibrates around 435 cm^−1^ [53].

#### 3.1.3. Surface Morphology Studies via AFM Investigations

**Figure 4:**
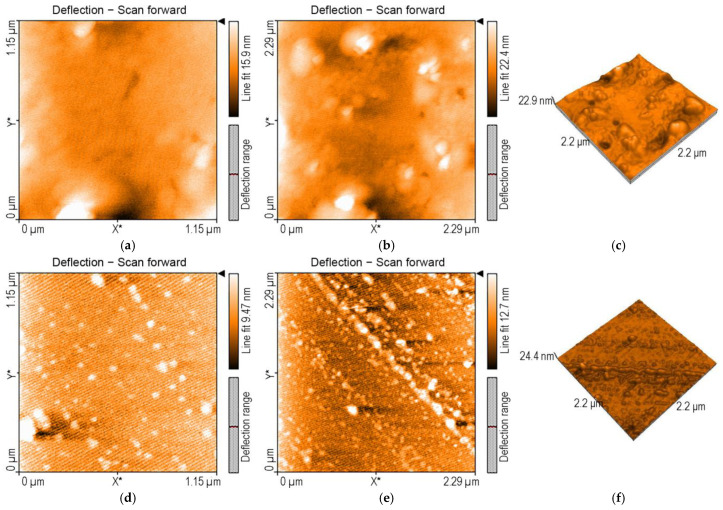
AFM images of (**a**–**c**) 3,4-diMeOPP base porphyrin and (**d**–**f**) Mn-3,4-diMeOPP, deposited from THF.

In AFM imaging, the porphyrin base, 3,4-diMeOPP, deposited from THF (Figure 4a–c), shows large, unevenly distributed haystack type formations with a medium dimension of 550 nm, organized in H- and J-type aggregates, with a height distribution from 6 to 12 nm.

Regarding Mn-3,4-diMeOPP chloride deposited from THF, AFM images show haystacks aggregates, finally self-orientated and self-organized by the J-type process in linear rows, as can be seen in Figure 4d–f with a height distribution between 6.5 and 10.9 nm.

The value of nanorugosity for Mn-3,4-diMeOPP is Sa= 1.07 pm. The value of the highest peak, Sp, is 4.18 nm, whereas for the lowest valley, Sv, at −5.75 nm, it is slightly lower.

### 3.2. UV–Vis Method for TAA Detection/Quantification Using Mn-3,4-diMeOPP as Sensitive Material: AFM Morphological Study of Surfaces After TAA Detection

**Figure 5:**
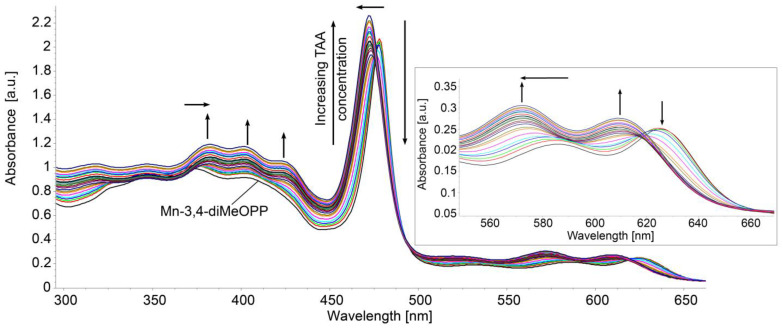
The changes in UV–Vis spectra of Mn-3,4-diMeOPP during the addition of thioacetamide in THF/water solution 1/1 *v*/*v*. The changes in Q band intensity and allure are highlighted in detail.

As can be seen in Figure 5, the successive adding of TAA solution in water to a diluted Mn-3,4-diMeOPP solution in THF (c = 3.180 × 10^−5^ M) leads to significant changes regarding the position of the bands in the UV–Vis spectrum. Thus, the Soret band initially located at 478 nm suffers a hypsochromic shift, to 472 nm, associated with a hyperchromic effect. This behavior confirms an interaction between the manganese atom in the center of the porphyrin ring with the nitrogen atom from TAA and the reduction of Mn(III) to Mn(II), as previously presented in the literature [48,54]. Other important modifications occur on the Q bands. Thus, the Q band located initially at 625 nm is shifted to 610 nm, and the one located at 586 nm is hypsochromically shifted to 572 nm. The modifications are accompanied with the formation of an isosbestic point, at 619 nm. All these modifications indicate that the formed Mn(II) complex has a square pyramidal geometry and the metal is out of plane [48]. On the hyper bands that are characteristic of manganese porphyrins, the presence of an increasing absorption peak is noticeable at 424 nm, as well as the bathochromic shift of the hyper band from 373 nm to 382 nm.

**Figure 6:**
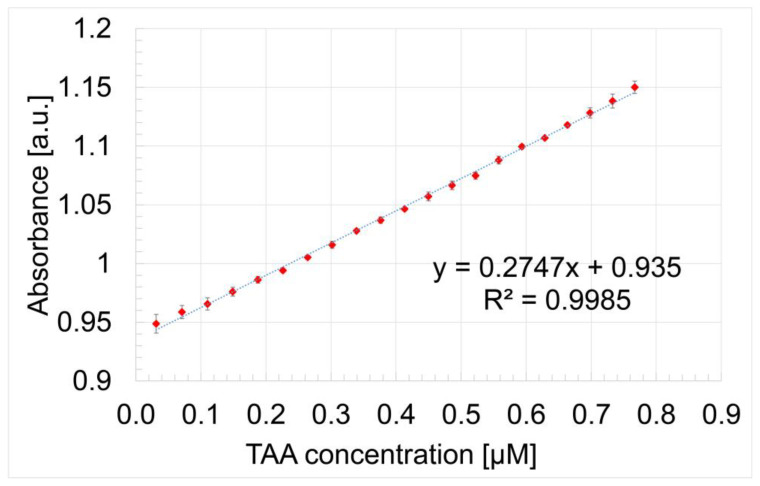
The linear dependence between the absorbance of Mn-3,4-diMeOPP measured at 401.5 nm and the TAA concentration.

In Figure 6, the capacity of Mn-3,4-diMeOPP to optically detect TAA is presented as a linear dependence of the absorbance of the metalloporphyrin read at 401.5 nm and the concentration of TAA in the range from 3.13 × 10^−8^ M to 7.67 × 10^−7^ M (0.031–0.767 μM), with a confidence coefficient of 99.85%. Error bars were calculated using the standard deviation function in Microsoft Excel based on three experimentally obtained data sets.

The slope of the calibration curve represents the sensitivity of the TAA detection method, defined as the absorbance of TAA concentration [55].

The limit of detection (LOD) and limit of quantification (LOQ) were calculated for TAA using Equations (1) and (2), respectively.

LOD = 3.3 × N/B(1)

LOQ = 10 × N/B(2)

where N is the standard deviation of the peak areas of the TAA and B is the slope of the corresponding calibration curve [56].

The calculated LOD for TAA detection using Mn-3,4-diMeOPP, determined by applying Equation (1), is 1.31 × 10^−8^ M. The limit of quantification calculated using Equation (2) is 4.36 × 10^−8^ M.

**Figure 7:**
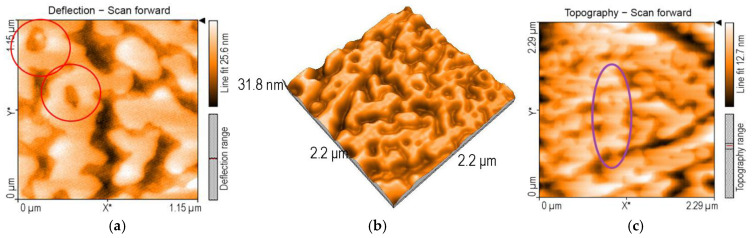
(**a**) 2D AFM image of Mn-3,4-diMeOPP after exposure to TAA and (**b**) 3D image and (**c**) topography of the same.

Analyzing the morphology of the surfaces by atomic force microscopy, after the detection of TAA using Mn-3,4-diMeOPP as sensitive material, two types of significant aggregations are noticed. A first form of aggregate, generated by both H- and J-types of aggregation, reveal donuts of a similar shape and size, with a diameter of around 250 nm (Figure 7a) and a wide span of heights, between 9 and 22 nm.

The 3D (2 × 2 micrometer) image (Figure 7b) shows these donuts linked in a complex labyrinth with large channels with valley depths of 7.16 nm. The donuts continue to self-aggregate, joining three units into helicoidal columns, to form mixing spindles (Figure 7c). The nanorugosity after TAA detection is higher than that for the Mn-3,4-diMeOPP, at a value of Sa = 2.21 pm. The value of the highest peak, Sp, is 7.98 nm, whereas that of the lowest valley, Sv, is −11.73 nm.

### 3.3. Detection of TAA Using Gold Colloidal Solution (AuNPs) as Sensitive Material: Study of Morphological Modifications to the Surfaces Before and After Exposure

**Figure 8:**
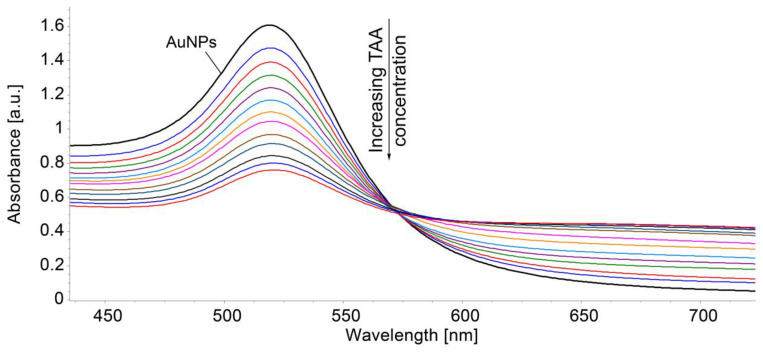
Overlapped UV–Vis spectra of TAA detection using gold colloid (maximum of absorption 522 nm).

Due to the high affinity between sulfur and gold [57,58], we tried to accomplish the detection of TAA by using AuNPs in order to compare the two UV–Vis methods. The overlapped UV–Vis spectra of AuNPs produced during the detection of TAA are presented in Figure 8.

**Figure 9:**
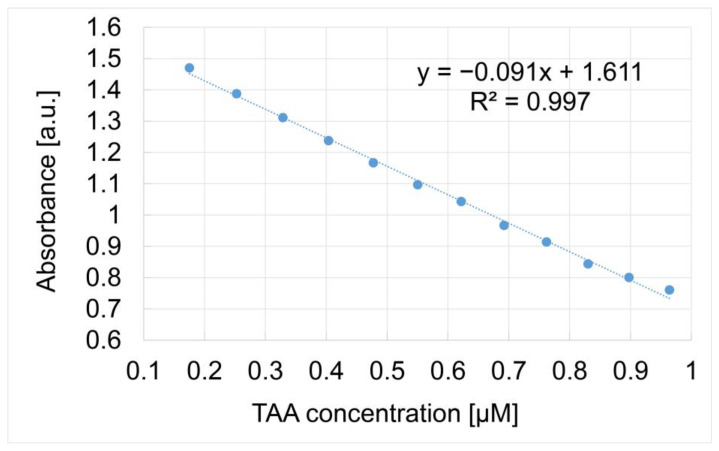
Linear detection of TAA in the 2–9.8 × 10^−7^ M concentration domain using gold colloid.

As can be noticed, the detection of TAA based on gold colloid is limited to a narrow field but is fairly accurate in the trace domain. The linear dependence between the absorbance of gold plasmon read at 522 nm and the concentration of TAA in the range 2–9.8 × 10^−7^ M is presented in Figure 9.

In comparison with the UV–Vis method using a gold colloidal solution as the sensitive material, the method using Mn-3,4-diMeOPP is more accurate, more precise, and efficient for a larger domain, making it more suitable to meet TAA detection demands in the toxicity- and illness-generating concentration limits.

**Figure 10:**
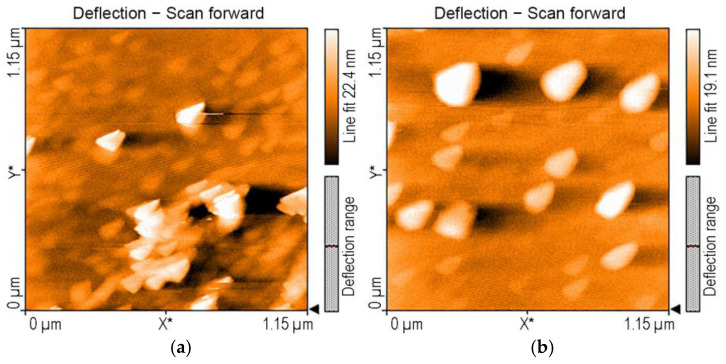
AFM 2D images of (**a**) AuNPs and (**b**) AuNPs after exposure to TAA.

Analyzing the AFM images before and after the exposure of AuNPs to TAA, the main difference between AuNP isosceles triangular aggregates, with dimensions varying from 30 to 50 nm (Figure 10a), and AuNP aggregates after TAA detection (Figure 10b) is that the latter are equilateral triangles, growing in size to about 150 nm. Regarding the nanorugosity, it decreases after TAA recognition to Sa = 0.98 nm compared to the value of Sa = 1.61 nm in bare AuNPs.

### 3.4. Generation of the Mn-3,4-diMeOPP-AuNP Complex

**Figure 11:**
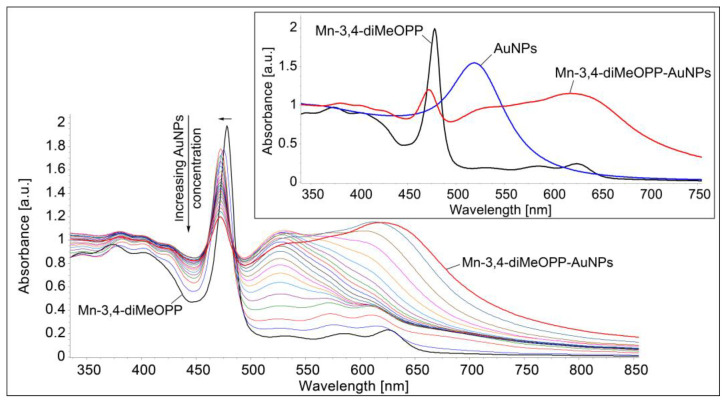
UV–Vis monitoring of Mn-3,4-diMeOPP-AuNP complex formation in THF/water. In detail, the superposed UV–Vis spectra of Mn-3,4-diMeOPP, AuNPs, and the Mn-3,4-diMeOPP-AuNP complex.

The gold nanoparticles are negatively charged, so they interact with the positively charged manganese ion located in the porphyrin macrocycle. Therefore, the Soret band undergoes a hypsochromic shift, from 478 nm to 472 nm (Figure 11). Under the influence of increasing gold colloid concentration, the hyperchromic effect of the new band located at 618 nm can also be noticed, along with a considerable enlargement of the plasmonic band from 350 nm to 750 nm.

### 3.5. Detection of TAA Using Mn-3,4-diMeOPP-AuNP Complex as Sensitive Material and AFM Analysis of Morphological Modifications

**Figure 12:**
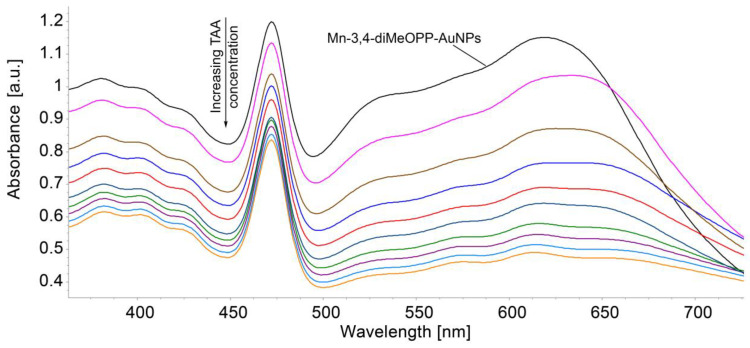
Overlapped UV–Vis spectra after the exposure of the Mn-3,4-diMeOPP-AuNP complex to TAA in THF/water.

**Figure 13:**
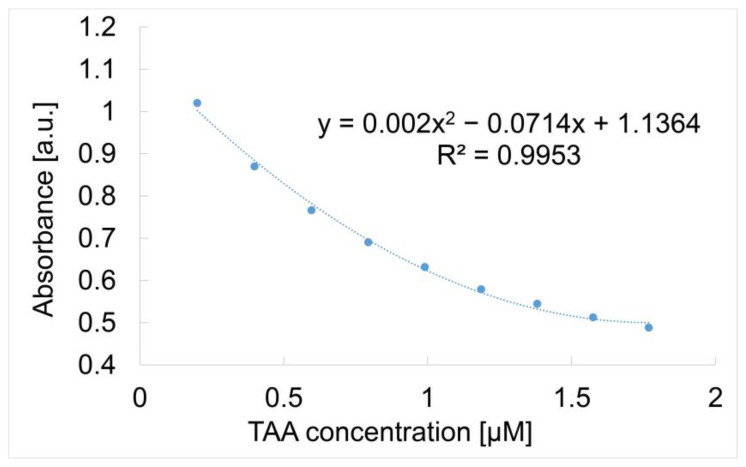
Dependence between the absorbance of Mn-3,4-diMeOPP-AuNP plasmonic complex read and 620 nm, and the TAA concentration in the range 1.99 × 10^−8^ M–1.76 × 10^−7^ M.

The method of detection was the same as that already presented in Materials and Methods Section 2.3. The composition of the hybrid solution containing both the metalloporphyrin and the gold colloidal solution is in the molar ratio Mn-3,4-diMeOPP/AuNPs = 1/4. The superposed UV–Vis spectra are shown in Figure 12. It can be seen that the increasing concentration of TAA leads to a decrease in the absorbance of the complex plasmon material. Nevertheless, the dependence between the absorbance and the TAA concentration is a second-order polynomial (Figure 13), nevertheless showing very good sensitivity.

Since the data points regarding the dependence between the concentration of TAA and absorbance response of the Mn-3,4-diMeOPP-AuNP complex could represent either a linear calibration model or a polynomial one, a Mandel test was conducted using the car package in R, which is presented in Supplementary Materials (Figures S1 and S2).

An explanation for this behavior could be the following: gold nanoparticles enhance optical signals through plasmonic effects, but the signal does not increase linearly due to saturation at high concentrations. On the other hand, gold nanoparticles may aggregate, altering the signal non-linearly. Binding between the analyte and the sensitive substance may follow complex, multi-step kinetics. Optical responses can deviate from linearity due to scattering or resonance. Another argument is that the gold from the Mn-porphyrin–gold complex has strong affinity to the sulfur atom from TAA, whereas the Mn-porphyrin interacts with the nitrogen atom from TAA.

**Figure 14:**
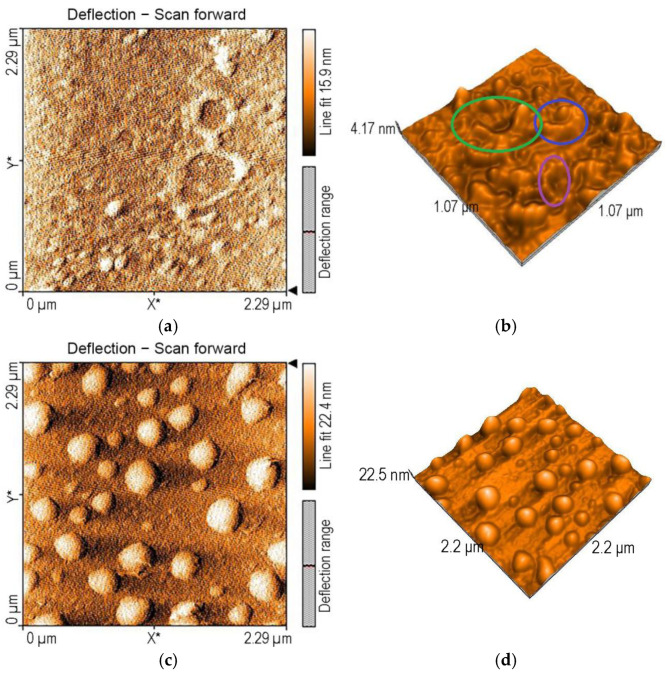
AFM images of (**a**,**b**) the Mn-3,4-diMeOPP-AuNP complex deposited from THF/water and (**c**,**d**) the Mn-3,4-diMeOPP-AuNP complex after exposure to TAA.

The surface morphology of the complex of Mn-3,4-diMeOPP and AuNPs (Figure 14a,b), analyzed by AFM, shows rings with different-sized diameters, ranging from 200 nm to 400 nm, presenting haystacks or even arches (segments of a circle), generated by joining together the two compounds. The nanorugosity, Sa, is in this case 1.58 nm, whereas the Sp is in this case 5.10 and the Sv is −8.55. After the complex interaction with TAA, the Sa increases to 1.73 nm, the Sp is also higher, reaching 6.09 nm, and the Sv is lower, at −9.09. This increase in rugosity might be proof of TAA interaction with the complex of Mn-3,4-diMeOPP and AuNPs.

In a peculiar way, after TAA detection, the aggregates between Mn-3,4-diMeOPP-AuNPs and TAA appear as large hemi-spheres or hemi-ovoid structures, very regular in shape and in size (300 nm in diameter) (Figure 14c,d). We observed this dome type of aggregation in previously published papers [59], and it is based on the dome distortion of the macrocycle due to the peripheral substituents or the metal center. The dome distortion has effects on the redox potentials, basicity, reactivity, catalytic activity, and coordinative abilities [60,61].

### 3.6. TAA Concentration Domain Complementarity for the Three Tested Sensitive Materials

**Figure 15:**
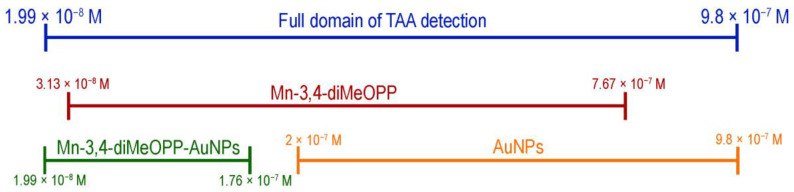
The TAA detection concentration range covered by the three materials used in this study. The three sensors are both overlapping and complementary.

Comparing the obtained data (Figure 15) for the tested sensitive materials, it can be concluded that the largest TAA concentration domain detectable in a linear fashion is provided when using Mn-3,4-diMeOPP alone, despite the fact that gold nanoparticles provide an increased sensitivity but in a very narrow concentration domain. The Mn-3,4-diMeOPP-AuNP complex also covers an extended concentration domain in the trace region, but the dependence between the absorbance and the TAA concentration is polynomial (Figure 13), and therefore the calibration of the method is more difficult.

### 3.7. Impact of Potential Interfering Species on TAA Detection Based on Mn-3,4-diMeOPP

As Mn-3,4-diMeOPP presented the best results in the quantification of TAA among all the sensitive materials tested in this paper, we only performed the interference experiments for this compound.

**Figure 16:**
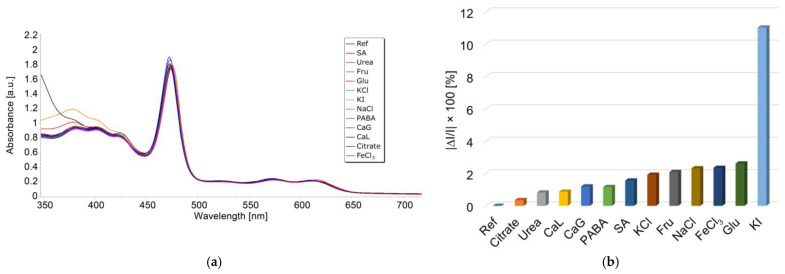
(**a**) Overlapped UV–Vis spectra representing the influence of diverse interfering species: citric acid (citrate), urea (urea), calcium lactate (CaL), calcium gluconate (CaG), para-aminobenzoic acid (PABA), salicylic acid (SA), KCl, fructose (Fru), NaCl, FeCl_3_, glucose (Glu), KI. (**b**) The graphical representation of average percentage errors for TAA optical detection using Mn-3,4-diMeOPP, introduced by different interferences.

The effects of interfering species usually present together with TAA in the target media are the following: citric acid (citrate), urea (urea), calcium lactate (CaL), calcium gluconate (CaG), para-aminobenzoic acid (PABA), salicilic acid (SA), KCl, fructose (Fru), NaCl, FeCl_3_, glucose (Glu), and KI; they are presented in Figure 16a.

To a freshly prepared solution of 2.8 mL of Mn-3,4-diMeOPP in THF, 0.05 mL of TAA (c = 1 × 10^−^⁵ M) was added, followed by the addition of 0.1 mL of each interfering solution. The concentration of the interfering species in this investigation was 100 times higher than that of the thioacetamide. The samples were stirred for 1 min, and UV–Vis spectra were recorded. Each measurement was performed in triplicate.

The average percentage errors for TAA detection were calculated according to Equation (3):

|ΔI|/I × 100(3)

where I represents the absorbance of the sample containing TAA.

|ΔI| is the difference in the module between the absorbance of TAA and of each studied interference specie, as displayed in Figure 16b.

The interference species do not generate significant errors in the determination of TAA (with each tested compound introducing an error smaller than 2.6%) excepting the iodide anion, the presence of which is totally undesired. The I^−^ ion severely interferes with the TAA quantifications (errors higher than 10%).

### 3.8. Proposed Mechanism for the Recognition of TAA by Mn-3,4-diMeOPP

**Figure 17:**
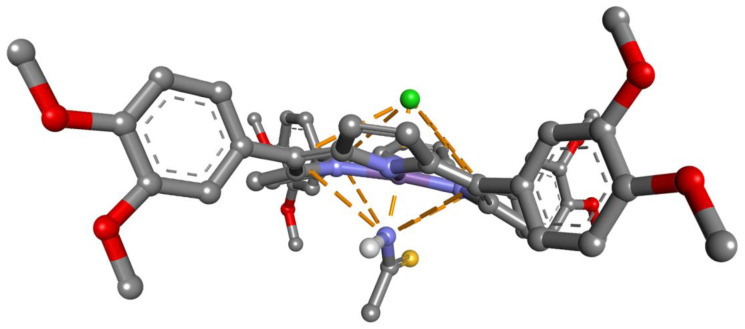
Proposed mechanism of interaction between Mn-3,4-diMeOPP and TAA. Atom color coding: carbon, black; oxygen, red; nitrogen, blue; sulfur, yellow; chlorine, green; manganese, purple.

The modifications of the UV–Vis spectra and of the surface morphology after the exposure of Mn-3,4-diMeOPP to TAA led us to presume a mechanism of recognition based on the interaction of the manganese ion toward the nitrogen atom in TAA [62], as presented in Figure 17. To underline this assumption, previous papers reported that Co(III)- and Mn(III)-porphyrins could axially coordinate amino/amido ligands and offer a “wheel-and-axle”-type shape for the supramolecular assemblies [62,63].

Starting with crystallographic data (entry 4123604, Crystallography Open Database) [64], the porphyrin structure was constructed by adding phenyl substituents and a manganese ion (initially +1 charge), followed by geometry optimization using Schrodinger software [65]. Interaction analysis and visualization were carried out using Biovia Discovery Studio [66]. Due to electron delocalization towards the nitrogen atoms, the atoms of carbon from the *meso* position are positively charged. This electronic configuration might favor the generation of a grid of electrostatic interactions between the porphyrin and TAA [67], as shown in Figure 17.

In this case, substituent effects do not follow expectations based on the polarization of the *π* system; instead, a predominantly electrostatic model is found to be more appropriate [68]. With this larger *π*-system, the distortion of the porphyrin molecule [69] and the nature of the counterion also exert effects on the energetic level of interactions.

### 3.9. Comparison of the Efficiency of TAA Detection Methods

Critically analyzing the results obtained in our work with the results reported in the literature, presented in Table 1, it can be observed that the materials obtained in our study present better sensitivity to thioacetamide and a very good detection limit.

The optical detection presented in this paper by using Mn-3,4-diMeOPP as a sensitive material is efficient, with the second lowest reported detection limit (0.013 μM) and a fair detection range (3.13 × 10^−8^ M–7.67 × 10^−7^ M). This shows an excellent combination of precision, sensitivity, and selectivity for TAA trace detection. In comparison, methods based on fluorescence-ZnO quantum dots [39] have the best detection limit (0.00214 μM) but suffer from reproducibility difficulties and a narrow working range. Although the diamond-doped boron electrode voltammetry method [35] offers good stability and reproducibility, it is expensive, and mercury-based methods should be avoided due to toxicity problems.

## 4. Conclusions

A manganese(III)-porphyrin (Mn-3,4-diMeOPP) was synthesized, starting from 5,10,15,20-tetrakis-(3,4-dimethoxyphenyl)-21H,23H-porphyrin and manganese chloride. The new compound was characterized by means of UV–Vis, FT-IR spectrometry and AFM, followed by an investigation into its capacity to detect thioacetamide (TAA) by optical methods. Based on our previous experience, a complex of gold colloid and Mn-3,4-diMeOPP was also obtained and tested for thioacetamide detection, to verify whether it could leverage sensitivity and enlarge the detected concentration domain. The AuNPs alone can detect TAA, but the concentration domain was extremely narrow: 2–9.8 × 10^−7^ M. It can be concluded that Mn-3,4-diMeOPP is the most sensitive and selective material, as it can quantify TAA in the range 3.13 × 10^−8^ M–7.67 × 10^−7^ M, in a linear fashion, with a 99.85% confidence coefficient. The complex formed between Mn-3,4-diMeOPP and gold colloid was shown to be able to quantify TAA in the concentration domain of 1.99 × 10^−8^ M–1.76 × 10^−7^ M, giving the lowest LOD, but the dependence between the absorbance and the TAA concentration was polynomial, proving to be more difficult to apply to real sample determination. A potential mechanism for TAA detection based on Mn-3,4-diMeOPP is discussed based on computational modeling. As a result of the conjugated effects of the distorted porphyrin conformation and its electronic configuration, it is presumed that a network of electrostatic interactions between Mn-porphyrin and TAA took place.

## Figures and Tables

**Table 1:** Various methods reported in the literature for the detection of TAA using different sensing materials.

Sensitive Material/ Detection Method	Concentration Domain [μM]	Detection Limit [μM]	Advantages/Disadvantages	Ref.
Boron-doped diamond electrode/voltammetry	5–60	0.84	Good reproducibility and stability/expensive diamond electrode	[35]
Catechol/ voltammetry	5–125	2.02	High sensitivity, no cationic interferences	[36]
Differential pulse cathodic stripping voltammetry	−	0.022	Low detection limit/toxic mercury is used	[37]
Nanofibers composed of copper and aspartate/triboelectric nanogenerator (TENG-sensing)	1000–100,000	−	Creation of a triboelectric nanogenerator	[38]
ZnO quantum dots/ fluorescence	0–10	0.00214	Difficult to replicate, narrow concentration domain	[39]
Tris(2,2′-bipyridyl)ruthenium(II)/ electrochemiluminescence	10–1,000,000	0.035	Low detection limit, outstanding recoveries	[40]
AuNPs/optical detection	0.2–0.98	0.069	Detection in low field concentrations/very narrow concentration domain	This work
Mn-3,4-diMeOPP-AuNPs/optical detection	0.019–0.176	0.006	Trace field detection/polynomial dependence between the absorbance and the TAA concentration	
Mn-3,4-diMeOPP/optical detection	0.0313–0.767	0.013	Best performing of all reported sensors; an accurate, sensitive, and selective sensor for trace field detection	

## Data Availability

The data presented in this study are available on request from the first or the corresponding author.

## Supplementary Materials

The following supporting information can be downloaded at: https://www.mdpi.com/article/10.3390/mi16050574/s1, The Mandel test was performed according to literature [70,71,72]. Figure S1: Dependence between concentration of TAA and absorbance response of the Mn-3,4-diMeOPP-AuNP complex.; Figure S2: Residual plot.
