# Supplementary material for: UV–Vis Detection of Thioacetamide: Balancing the Performances of a Mn(III)-Porphyrin, Gold Colloid, and Their Complex for Selecting the Most Sensitive Material
Source: Micromachines (Basel). 2025 May 14;16(5):574. doi: 10.3390/mi16050574 (PMC12114081; doi:10.3390/mi16050574)
Supplement: Supplementary file 1 [file micromachines-16-00574-s001.zip › micromachines-3604316-supplementary.pdf]

Supplementary Material

UV-Vis Detection of Thioacetamide. Balancing between Performances of a Mn(III)-Porphyrin, Gold Colloid and their Complex for Selecting the Best Sensitive Material

Camelia Epuran[1], Ion Fratilescu[1,*], Ionela Fringu[1,*], Anca Lascu[1], Liliana Halip[1], Mihaela Gherban[2] and Eugenia Fagadar-Cosma[3,*]

[1] Institute of Chemistry "Coriolan Dragulescu", Mihai Viteazu Avenue 24, 300223 Timisoara, Romania; ecamelia@acad-icht.tm.edu.ro (C.E.); ionfratilescu@acad-icht.tm.edu.ro (I.F.); mcreanga@acad-icht.tm.edu.ro (I.F.); alascu@acad-icht.tm.edu.ro (A.L.); lili.ostopovici@acad-icht.tm.edu.ro (L.H.)

[2] National Institute for Research and Development in Electrochemistry and Condensed Matter, P. Andronescu Street, No. 1, 300224 Timisoara, Romania; mihaelabirdeanu@gmail.com (M.B.)

[3] Romanian Academy, School of Advanced Doctoral Studies of the Romanian Academy (SCOSAAR), Department: Institute of Chemistry "Coriolan Dragulescu", Mihai Viteazu Avenue 24, 300223 Timisoara, Romania; efagadar@yahoo.com (E. F.-C.)

* Correspondence: efagadar@yahoo.com (E. F.-C.); ionfratilescu@acad-icht.tm.edu.ro (I.F.); mcrean-ga@acad-icht.tm.edu.ro (I.F.)

Since the data points regarding the dependence between concentration of TAA and absorbance response of the Mn-3,4-diMeOPP-AuNPs complex could represent either a linear calibration model (blue) or a polynomial one (red) (Figure S1), a Mandel test was conducted using the car package in R [70-72] to statistically determine which model provides a better fit. The test yielded a p-value of 7.384 × 10^{-5}, which is well below the commonly used significance threshold of 0.05.

This result strongly suggests that the linear model is inadequate for describing the relationship between the variables. Therefore, a more complex model is needed to capture the structure in the data. A quadratic model, which includes a squared term to account for curvature, was subsequently fitted. This model showed a significantly better fit, supporting the presence of non-linearity in the calibration curve.

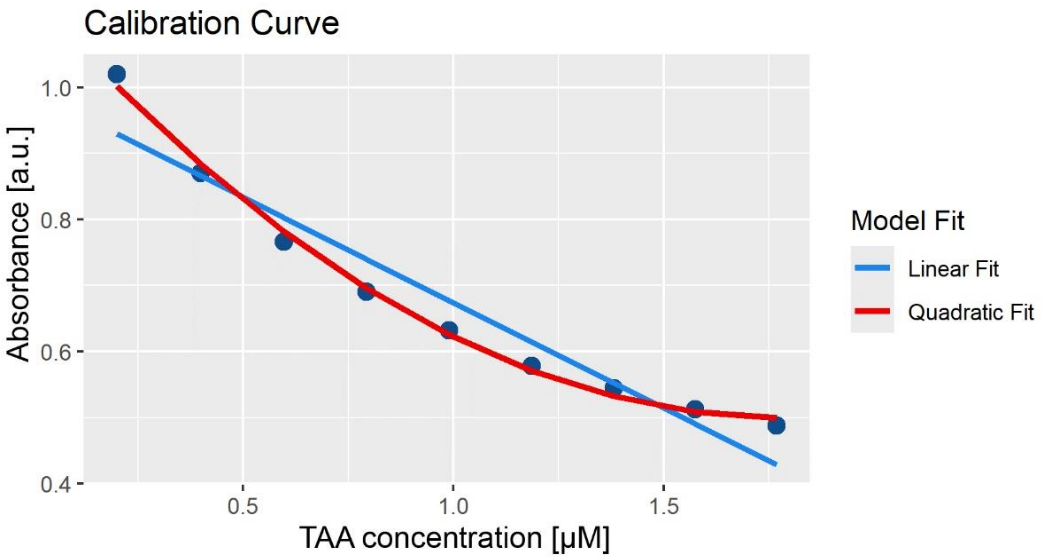

Figure S1. Dependence between concentration of TAA and absorbance response of the Mn-3,4-diMeOPP-AuNPs complex.

Additional evidence for the inadequacy of the linear model results from the examination of the residuals plot (Figure S2). The residuals—the differences between observed and predicted values—exhibit a systematic curved trend, rather than random scatter. Such a pattern is a classic indicator of model misspecification and confirms the presence of curvature in the data, further justifying the use of a polynomial model.

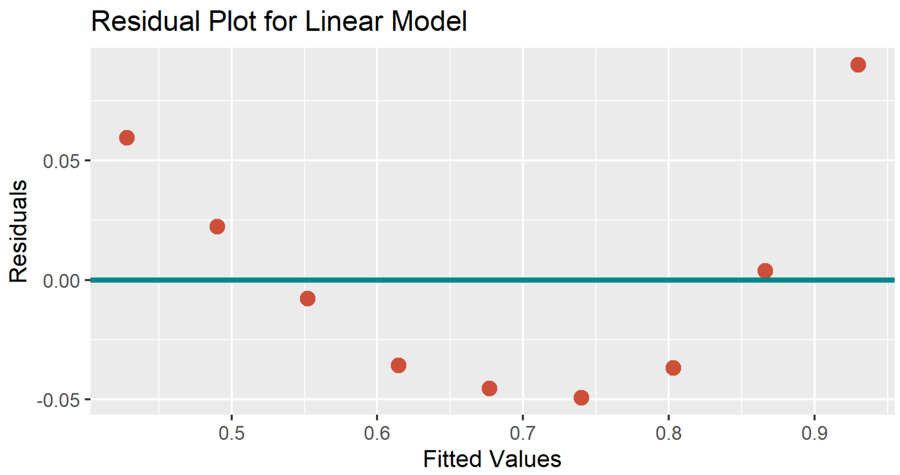

Figure S2. Residuals plot.

References

70. Fox, J.; Weisberg, S. *An R Companion to Applied Regression*3rd ed.; Sage Publications: Thousand Oaks, CA, USA, 2019; https://www.john-fox.ca/Companion/

71. Mandel, J. The validation of measurement through interlaboratory studies. *Chemometr. Intell. Lab. Syst*. **1991**, *11*, 109−119. https://doi.org/10.1016/0169-7439(91)80058-X.

72. Wilrich, P.-T. Critical values of Mandel's h and k, the Grubbs and the Cochran test statistic. *AStA-Adv. Stat. Anal*., **2013**, *97*(1), 1−10. https://doi.org/10.1007/s10182-011-0185-y.
